# Research on the protective effect of caffeic acid phenethyl ester on testicular damage caused by cisplatin

**DOI:** 10.3906/sag-2002-58

**Published:** 2020-12-17

**Authors:** Tayfun CEYLAN, Emin KAYMAK, Fazile CANTÜRK, Birkan YAKAN

**Affiliations:** 1 Department of Histology and Embryology, Faculty of Medicine, Erciyes University, Kayseri Turkey; 2 Department of Biophysics, Faculty of Medicine, Erciyes University, Kayseri Turkey

**Keywords:** Caffeic acid phenethyl ester, cisplatin, comet assay, rat, testes

## Abstract

**Background/aim:**

Cisplatin (CP), a chemotherapeutic drug, causes damage to spermatogenic serial cells, Sertoli cells, and Leydig cells in rat testicles. It was aimed to investigate the protective effect of caffeic acid phenethyl ester (CAPE), one of the active ingredients of propolis, in eliminating CP-induced testicular damage.

**Materials and methods:**

Group 1 (control group) was given physiological saline solution intraperitoneally (IP) throughout the experiment. Group 2 (CP group) was given a single dose of CP (7 mg/kg) IP on the day 7. Group 3 (CP + CAPE group), was given CAPE (10 µmol/kg/day) IP for 12 days and a single dose of CP (7 mg/kg) IP on day 7. Group 4 (CAPE group) was given CAPE (10 µmol/kg/day) IP for 12 days. On day 14 of the experiment, the rats were decapitated under xylazine and ketamine anesthesia and their testicles were removed. The sections obtained from the testicles were stained with hematoxylin-eosin and histopathological damage was evaluated. Malondialdehyde (MDA) levels, and superoxide dismutase (SOD) and catalase (CAT) enzymatic activities were measured in the testicular tissue samples. Testosterone (TES) levels were measured in the blood serum. The Johnsen testicular biopsy score (JTBS) was used to evaluate testicular tubules. DNA damage was evaluated in sperm samples taken from the ductus epididymis using the comet assay technique.

**Results:**

In Group 2, which was given CP, the testicles were severely damaged. It was observed that histological damage was reduced in the testes by administering CAPE in Group 3. Moreover, according to the JTBS, the value was significantly higher in the testicular tubules (P < 0.05). Moreover, the MDA level decreased in Group 3. However, the SOD, CAT, and TES levels increased in Group 3. DNA damage also decreased significantly in Group 3 when compared to Group 2 (P < 0.05).

**Conclusion:**

The results showed that CAPE may be protective against damage caused by CP in the testicles of rats.

## 1. Introduction

Chemotherapy drugs are used in the treatment of various types of cancers. Their toxicities and minor risks are often overlooked as a result of their potential usefulness in treatment. In particular, testicular cells have been selected as targets by chemotherapeutic agents, as they enter various mitotic, meiotic, and morphological processes and therefore they are easily damaged [1]. Cis-diamminedichloroplatinum (II) or cisplatin (CP), a highly effective antineoplastic DNA alkylating agent, has been used to treat a wide variety of solid tumors, such as those in the testicles, ovaries, breast, lungs, bladder, head, and neck [2]. However, the use of the drug is limited due to side effects, such as testicular toxicity, cachexia, and testicular damage [2,3]. When CP is administered at a high cumulative dose, it can result in permanent azoospermia and then infertility in patients [3]. CP interacts with DNA to create cross-link and intra-chain cross-links. Since the DNA modified by CP cannot be sufficiently renewed, the DNA damage initiates apoptosis [4].

Caffeic acid phenethyl ester (CAPE) is one of the active ingredients of the sharp and fragrant propolis substance found in the extract collected by bees from plants. CAPE has been used as a folk remedy for many years [5]. Antimicrobial, antiinflammatory, immunomodulatory, antimutagenic, antioxidant, and anticarcinogenic effects of propolis have been demonstrated by various studies. CAPE, one of the active ingredients of propolis, and has an important place in these beneficial effects [6,7]. Although CAPE is a pharmacologically safe compound, it also reduces lipid peroxidation and stimulates antioxidant enzyme activity [8].

In this study, the effects of CAPE on testicular damage, DNA fragmentation, and antioxidant capacity mechanisms in CP-induced testes injury were evaluated.

## 2. Material and methods

### 2.1. Animals and drug administration

The study protocol was accepted by the Experimental Animal and Local Ethics Committee of Erciyes University (decision no: 15/59). This study was supported by the Erciyes University Scientific Research Project Unit under number TYL-2015-5948. Herein, 38 male adult Wistar albino rats, weighing between 150 and 220 g, and aged between 8 and 10 weeks, were supplied by the Experimental Animal Laboratory of Erciyes University. The rats were housed at 20–22°C under a 12:12 light/dark photoperiod and fed ad libitum.

The animals were randomly divided into 4 groups. Group 1 was given a physiological saline solution (1 mL/ kg/day) intraperitoneally (IP) throughout the experiment. Group 2 (CP group) was given a single dose of CP (Koçak Pharma, İstanbul, Turkey) (7 mg/kg) IP on day 7 of the experiment. The dose of CP was selected according to a previous study that demonstrated significant testicular toxicity in rats [9]. Group 3 (CP + CAPE group) was given CAPE (Sigma-Aldrich, St. Louis, MO, USA) (10 µmol/kg/day) IP for 12 days and single dose of CP (7 mg/kg) IP on ​​day 7 of the experiment. The dose of CAPE was selected based on the results of recent studies where the antioxidant and antiinflammatory action of this agent was apparent [10,11]. Group 4 (CAPE group) was given CAPE (10 µmol/kg/day) IP for 12 days. At the end of the study, rats were decapitated after intraperitoneal ketamine (75 mg/kg) xylazine (10 mg/kg) anesthesia, and the testis tissues were rapidly removed. The sections obtained from the testicles were stained with hematoxylin-eosin (H&E) and the histological damage was evaluated. Malondialdehyde (MDA) levels and superoxide dismutase (SOD) and catalase (CAT) enzymatic activities were measured in the testicular tissue samples. Testosterone (TES) levels were measured in the blood serum. The Johnsen testicular biopsy score (JTBS) was used to evaluate the testicular tubules. DNA damage was evaluated in the sperm samples taken from the ductus epididymis using the comet assay technique.

### 2.2. H&E staining

First, the 5-µm sections taken from the paraffin blocks were spread out on slides. Standard histological methods were applied to the prepared slides. The paraffin was removed with xylol and passed through a graded alcohol series and diluted. The sections were stained with H&E to observe the general histological structure. The sections were examined after passing through an increasing alcohol series and xylene. H&E staining was purchased from Nanotek Lab (Kayseri, Turkey). The slides were then examined under an Olympus BX51 microscope (Tokyo, Japan).

### 2.3. The JTBS

According to this scoring, after any event causing damage, the distribution of the cells in the testicle tubules disappears progressively, following a certain order. The JTBS was used to assess the degree of this damage to the tubules [12]. The results of the histological examinations were evaluated by 2 specialist histologists at the Department of Histology and Embryology of the Erciyes University Medical School. Evaluation was made by examining 20 different tubules from 10 different randomly selected preparations from each group. For each group, 200 tubules were evaluated separately and the average JTBS was calculated. IBM SPSS Statistics for Windows 21.0 (IBM Corp., Armonk, NY, USA) was used for statistical comparisons between the groups. The data obtained were processed on a form that had been prepared previously (Table 1).

**Table 1:** Johnsen testicular biopsy score.

Score	Histological findings	Score	Histological findings
1	No cells in the tubular section	6	There were few spermatids (5/tubule)
2	There were only Sertoli cells	7	There were too many spermatids without any sign of difference
3	Germ cells were just as spermatogonium	8	Late spermatids without mature spermatozoa
4	There were few spermatocytes (5/tubule)	9	There were few spermatozoa (5/tubule)
5	There were too many spermatocytes	10	Exact spermatogenesis was present with a large number of spermatozoa

### 2.4. Biochemical analysis

Biochemical analysis was performed according to the manufacturer’s instructions of the enzyme-linked immunosorbent assay (ELISA) kit. The study was conducted at the Department of Histology and Embryology Laboratory of the Erciyes University Medical Faculty, using commercial YH Biosearch Laboratory kits (Shanghai, China) obtained from Molgen Biotechnology Lab (Esenler, İstanbul, Turkey), comprising rat malondialdehyde ELISA kit, catalogue number: YHB0708Ra; rat super oxidase dismutase ELISA Kit, catalogue number: YHB1021Ra; rat catalase ELISA kit, catalogue number: YHB0207Ra; rat testosterone catalogue number: 201-11-5126 ( Shanghai Sunred Biological Technology). The results of the tissue samples were given as nmol/mg SOD for the MDA and ng/mg protein for the CAT, and the results of the serum samples were given as nmol/mL for the TES.

### 2.5. Comet assay technique

Diluted sperm samples extracted from the epididymis were centrifuged at 300 *g* for 10 min at 4 °C. The supernatant was removed and the remaining sperm cells were washed with Ca+2 and Mg+2 free phosphate buffered saline. Sperm with fragmented DNA were determined using the single-cell gel electrophoresis (comet) assay, which was generally performed at high alkaline conditions. The images of 100 randomly chosen nuclei from the sperm sample of each animal were visually analyzed and sperm with fragmented DNA were counted. Observations were made at a magnification of 400× using an Olympus fluorescent microscope (Olympus Corporation, Tokyo, Japan). The damage was determined from the broken DNA tail that migrated from the sperm head and caused the comet. Cells with tails were evaluated as damaged [13].

### 2.6. Statistical analysis

All statistical analyses were conducted using SPSS. The Kolmogorov-Smirnov test was used to identify normal distribution of the data. In the case of normal distribution, quantitative variables were compared using 1-way ANOVA and the Tukey post hoc test. Descriptive statistics were shown as the mean ± SD. Statistical significance was accepted as P < 0.05.

## 3. Results

### 3.1. Light microscopic examination

**Figure 1:**
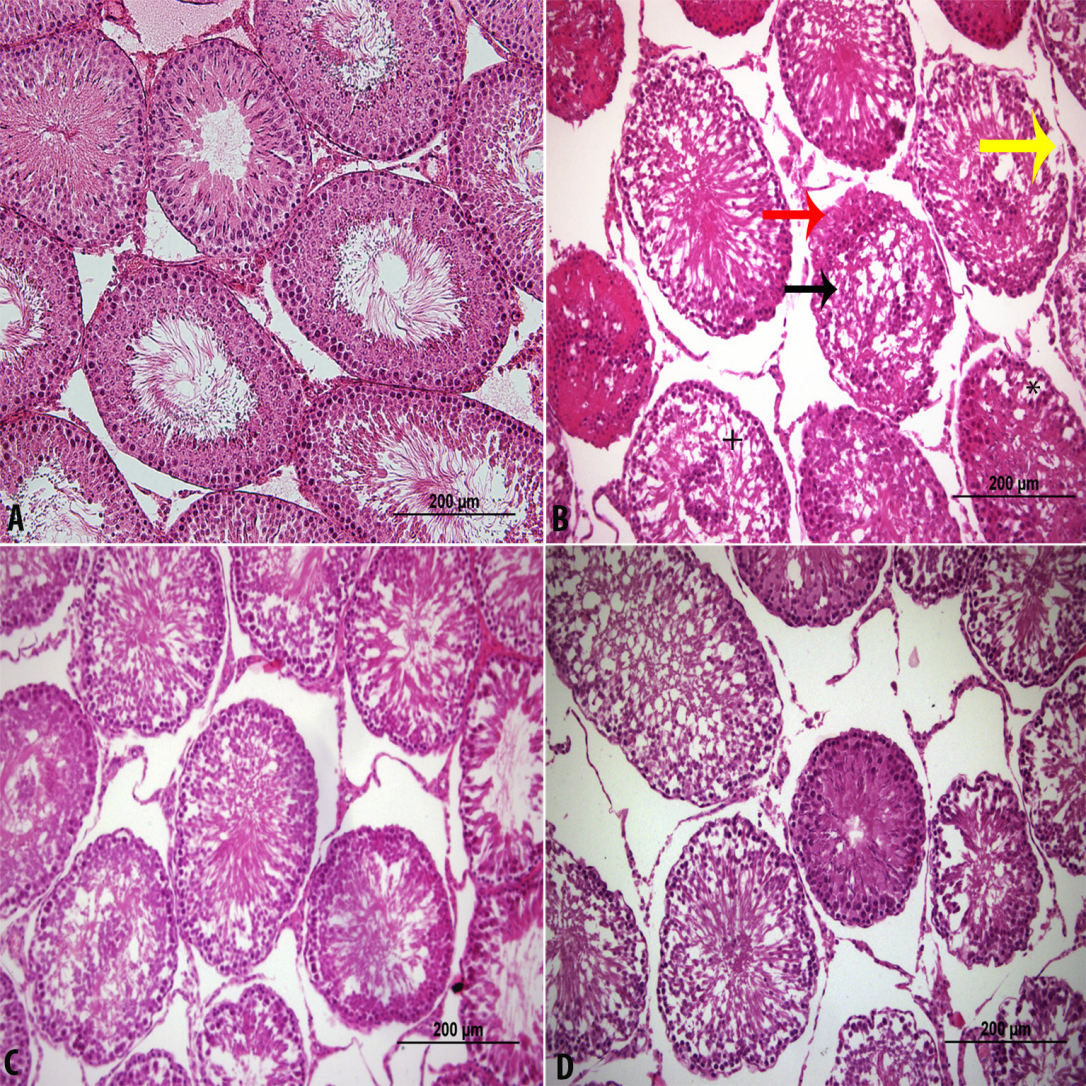
Control (A), CP (B), CP + CAPE (C), and CAPE (D) groups (H&E staining, × 200). * Shows intratubular edema. → Shows unspecific spermatogenic series cells. → Diffused basement membrane. → Impaired fibromuscular space. + Shows tailed spermatozoa in the lumen.

**Figure 2:**
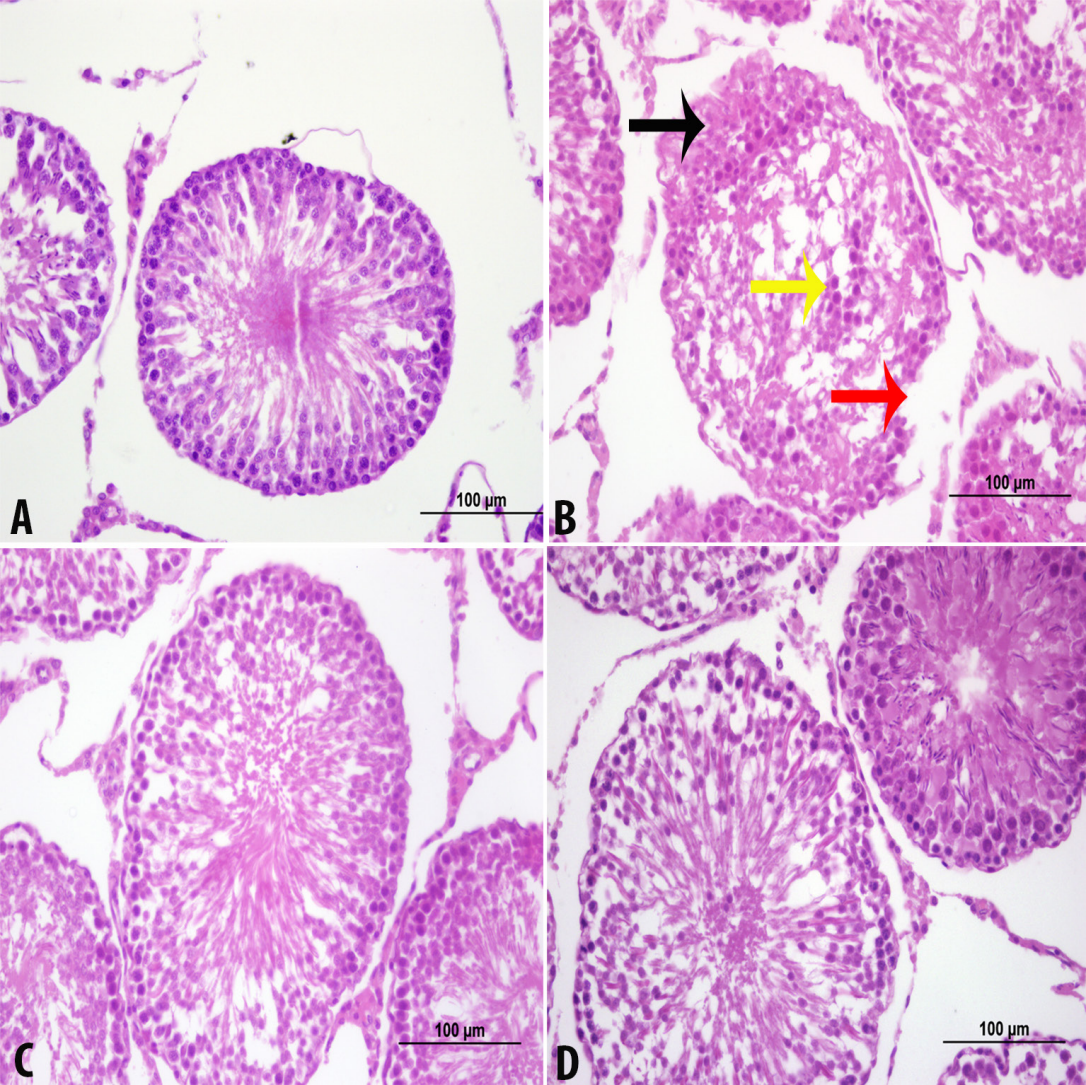
Control (A), CP (B), CP + CAPE (C), and CAPE (D) groups (H&E staining, × 400). → Impaired fibromuscular space. → Shows unspecific spermatogenic series. cells. → Diffused basement membrane

In Group 1, the testicles appeared to have normal histological features. The H&E staining images of Group 1 are shown in Figure 1A. In Group 2, there were intercellular spaces and local cell loss in areas near the lumen. The fibromuscular area was observed to be impaired. In some areas, basement membrane losses were observed. In the sections, deterioration in the basement membranes of the tubule seminar counters was observed. However, the nuclei of the cells in the lumen were evident, but they had no tail structures. Leydig cells in the interstitial space were partially damaged. Interstitial edema and cellular spaces were observed in the cell stages that should have belonged to spermatid serial cells. H&E staining images of Group 2 are shown in Figure 1B. Tubules were observed to be close to normal in Group 3. There were morphological signs of improvement in the Leydig cells in the interstitial space. In the sections, the seminiferous tubules were close to normal. Spermatogenic serial cells were significantly close to normal. The basement membrane was properly observed. H&E staining images of Group 3 are shown in Figure 1C. In Group 4, the stesticular histology was observed as close to that of Group 1. The H&E staining images of Group 1 are shown in Figure 1D. In addition, H&E staining images of all of the groups are shown in Figure 2. Figure 2A shows the normal seminiferous tubule structure. Figure 2B shows impaired fibromuscular space, unspecific spermatogenic series cells, and diffused basement membrane. A tubule structure that is close to normal is shown in Figures 2C and 2D.

### 3.2. JTBS results

The germinal epithelium in the seminiferous tubules was evaluated with the JTBS. According to these results, the biopsy score was decreased in Group 2 when compared to Group 1, and it was statistically significant (P < 0.05). The biopsy score in Group 3 increased numerically when compared to Group 2, and this increase was statistically significant (P < 0.05). In Group 4, the biopsy score was decreased when compared to Group 1. This decrease was statistically significant, although not as significantly as in Group 2 (P < 0.05). The JTBS results are shown in Table 2.

**Table 2:** Johnsen testicular biopsy score results.

	JTBS
Group 1 (n = 8)	8.12 ± 0.647
Group 2 (n = 10)	4.979 ± 0.983*
Group 3 (n = 10)	6.483 ± 0.656**
Group 4 (n = 10)	5.521 ± 0.453
P-value	<0.05

Values are given as the mean ± SD. P < 0.05. *Compared to Group 1, P < 0.05. **Compared to Group 2, P < 0.05.

### 3.3. Biochemical results

The MDA level, and CAT and SOD enzyme activities were evaluated in the testicular tissue using the ELISA technique. Moreover, the TES level was evaluated in the blood serum. When the analyses for CAT were evaluated, the activity in Group 2 was significantly decreased compared to Group 1. Moreover, in Group 3, the activity was higher than in Group 2. These values ​​were not statistically significant (P > 0.05). The SOD activity in Group 2 was lower than that in Group 1. However, the SOD activity in Group 3 was higher than that in Group 2. These values ​​were not statistically significant (P > 0.05). When the analyses for the MDA were evaluated, the level in Group 2 was higher than in Group 1, and the level Group 3 was lower than that in Group 2. These values ​​were not statistically significant (P > 0.05). When the analyses for the TES were evaluated, the level in Group 2 was lower than in Group 1, and the level in Group 3 was higher than that in Group 2. These values ​​were not statistically significant (P > 0.05). The ELISA technique results, in which the biochemical data were evaluated, are shown in Table 3.

**Table 3:** Biochemical data of the experimental groups.

	CAT	SOD	MDA	TES
Group 1 (n = 8)	19.015 ± 8.404	1.222 ± 0.949	0.84 ± 0.206	23.06 ± 14.22
Group 2 (n = 10)	14.299 ± 6.645	0.978 ± 0.443	0.852 ± 0.12	18.56 ± 2.81
Group 3 (n = 10)	22.321 ± 10,01	1.526 ± 0.765	0.811 ± 0.09	26.99 ± 11.15
Group 4 (n = 10)	19.872 ± 16.041	1.615 ± 0.439	0.826 ± 0.087	26.85 ± 10.26
P-value	>0.05	>0.05	>0.05	>0.05

Values are given as the mean ± SD. P < 0.05.

### 3.4. Comet assay technique results

**Figure 3:**
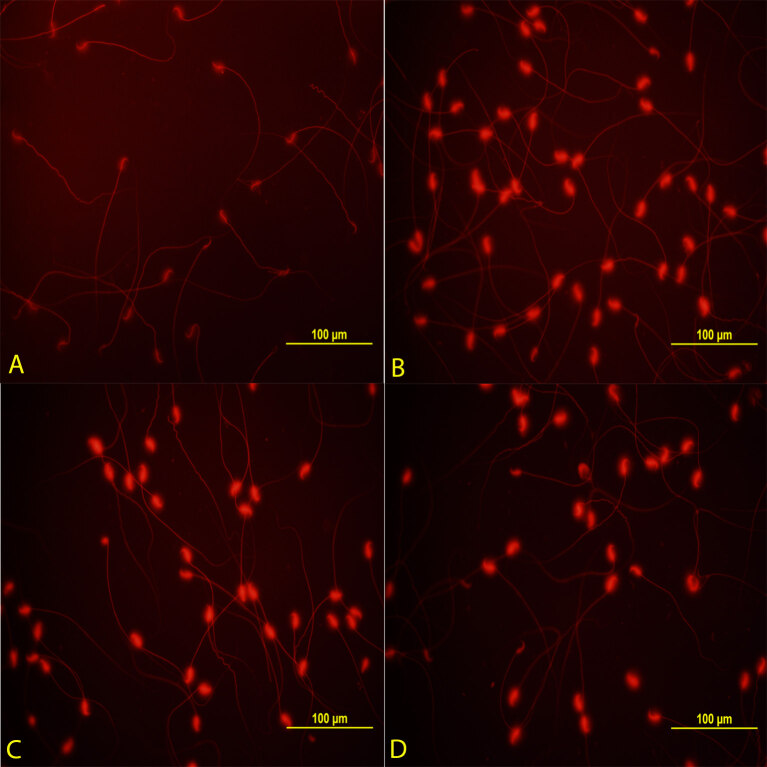
Control group (A); shows DNA undamaged sperm cells. CP (B), CP + CAPE (C), and CAPE (D) groups; shows DNA damaged sperm cells. (Ethidium bromide staining, × 400).

The microphotographic appearance of the fragmented DNA sperm and the head and tail DNA numbers in all of the groups are presented in Table 4, respectively. According to the data obtained as a result of measuring the microphotographic images, decreasing the amount of head DNA or increasing the amount of tail DNA resulted in DNA fragmentation in the cell. Only CP administration significantly increased the sperm DNA fragmentation rate when compared to Group 1 (P < 0.05). The comet assay results of Groups 1 and 2 are shown in Figures 3A and 3B, respectively. The sperm DNA fragmentation rate decreased significantly (P < 0.05) in Group 3 when compared to Group 2. The comet assay results of Groups 2 and 3 are shown in Figures 3B and 3C, respectively. Although the CAPE application alone increased the rate of sperm DNA fragmentation when compared to Group 1, this increase was less than that of Group 2 (P < 0.05). Comet assay results of the Groups 1 and 2 are shown in Figure 3D.

**Table 4:** Comet assay results.

	Head DNA	Tail DNA
Group 1 (n = 8)	98.969 ± 0.6	1.031 ± 0.6
Group 2 (n = 10)	79.28 ± 4.584 *	20.706 ± 4.579 *
Group 3 (n = 10)	88.383 ± 2.133 **	11.616 ± 2.133 **
Group 4 (n = 10)	84.733 ± 3.398	15.266 ± 3.398
P-value	<0.05	<0.05

Head and tail DNA values were evaluated separately. Values are given as the mean ± SD. P < 0.05. * Compared to Group 1, P < 0.05. ** Compared to Group 2, P < 0.05.

## 4. Discussion

CP is a drug used to treat some types of cancer in chemotherapy [14]. CP is an organic platinum-derived drug that has a structure unlike that of any other antineoplastic drug. Testicular, ovarian, and bladder carcinomas, in particular, are sensitive to CP-based chemotherapy [1]. CP, a chemotherapeutic drug, causes a number of side effects, including cachexia and testicular damage [3]. In addition to side effects, such as nephrotoxicity, neurotoxicity, and gastrointestinal irritability, it has been reported that it causes azoospermia, oligospermia, and infertility [1]. CP therapy may result in permanent azoospermia, and subsequently, infertility, in patients receiving a high cumulative dose [3].

Testicular damage due to CP occurs directly on spermatogenic cells and Sertoli cells. It also causes dysfunction in Leydig cells. It has been reported that sperm production, seminiferous tubule diameter, and intratesticular TES decreased and terminal deoxynucleotidyl transferase dUTP nick end labeling-positive cells increased in seminiferous tubules in CP-treated rats [14]. The most important point in cancer chemotherapy with CP is protection against side effects [15].

Soni et al. applied CP in testicular tissue and reported abnormal histopathological lesions in germinal cells, Sertoli, and Leydig cells [16]. Soni et al. demonstrated in another study the degeneration and disorganization of germinal, Sertoli, and Leydig cells in their 10 mg/kg CP treatment group [17]. However, a decrease in spermatogenic cell density was reported. They showed that the JTBS decreased significantly in their CP group. At the same time, there was a significant increase in MDA levels in their CP-treated group. In the current study, treatment with CP increased the level of MDA when compared to Group 1. Moreover, treatment with CP suppressed the antioxidant activity by lowering the CAT and SOD levels. Although the biochemical parameters showed tissue damage, this damage was not statistically significant.

In the current study, a single dose of CP (7 mg/kg) caused significant damage in rat testes. This damage was mostly in the spermatogenic series cells, deterioration in the sequence of series cells, loss in basement membranes, and in the areas near the lumen, (spleen-free) spermatozoon. According to the histopathological evaluations, the degree of damage to the primary and secondary spermatocytes was observed in the spermatogenic cell series. Cells in the lumen of the seminiferous tubule were nucleated, but they lost their tails. Leydig cells in the interstitial area were also partially damaged. The order of the series of spermatogenic cells should not be differentiated. The results obtained in the current study were compatible with studies in which CP-induced testicular damage was created. The JTBS measurements suggested significant damage to the seminiferous tubules with CP.

The comet assay method is an important method used to determine the level of damage to DNA [18]. Sarıözkan et al. reported that sperm DNA integrity was induced by Taxans, as observed via the comet assay technique. In their study, the comet assay technique was used to demonstrate that Docetaxel, Paclitaxel, and Docetaxel + Paclitaxel impaired sperm DNA fragmentation when compared to their control group [13]. In the current study, CP-induced DNA damage was observed in sperm using the comet assay technique.

Propolis is used by bees to cover holes and cracks in the hive, repair and paste combs, narrow the hive entrance, and facilitate defense. Propolis is a disinfectant material that provides disinfection of the honeycomb eyes [19].

CAPE is one of the active ingredients of the sharp and fragrant propolis substance, which is extracted from plants by bees, and has been used in alternative medicine for many years [5]. The antimicrobial, antiinflammatory, immunomodulatory, antimutagenic, antioxidant and anticarcinogenic effects of propolis have been demonstrated by various studies. Most of these effects were related to CAPE, one of the active substances of propolis [6,7]. CAPE has been shown to reduce lipid peroxidation and induce antioxidant enzyme activity, and in addition, it is a pharmacologically safe compound [8,20]. Moreover, CAPE has been shown to inhibit the growth of different types of transformed cells [5].

Ferreira et al. reported that they applied CAPE at doses of 1, 5, 20, 25, and 50 µmol against CP-induced neurotoxicity in their model via cell culture, and reported that the most effective concentration providing the highest protection against cell death was 10 µmol [10]. Abdel-Daim et al. reported that the combination of CAPE (10 µmol/kg) and betaine (250 mg/kg) against Abamectin-induced hepatoxicity and nephrotoxicity, which are commonly used as plant protection and insecticides, had a protective effect [11]. Ayla et al. showed that, in sperm samples incubated with CAPE, the DNA damage was decreased with low chromatin condensation when compared to samples not incubated with CAPE, and at the same time, incubation with CAPE reduced the MDA levels [21]. Yilmaz et al. investigated the anticlastogenic effect of CAPE on CP-induced chromosomal defects in rat bone marrow cells [22]. First, CAPE was applied (10 µmol/kg), and then 24 h following that, CP (5 mg/kg) was applied. The animals treated with a single-dose of CP were reported to exhibit both an abnormal chromosomal error and abnormal metaphase in their bone marrow cells. In rats treated with CP + CAPE, the total chromosomal error number and total abnormal metaphase ratio were reported to be significantly lower (P = 0.0001). Armağan et al. aimed to treat the oxidative stress caused by the administration of methotrexate (IP, 20 mg/kg) by administering CAPE (IP, 10 µmol/kg) [23]. In their study, they showed that the CAT activity of the testis in the methotrexate group was lower than that in the CAPE-treated group. It was also reported that the CAT activity in the methotrexate group was lower than that of the CAPE-treated group. This study showed that oxidative stress induced by methotrexate was reduced by applying CAPE [23].

In conclusion, studies have shown that CAPE has a therapeutic effect on many tissues. In the current study, CAPE, which is one of the active substances of propolis was applied to prevent the damage caused by CP in rat testis tissue. Improvement in the seminiferous tubules of the testicular tissues of rats treated with CAPE together with CP were observed. An increase in the number of Leydig cells in the interstitial area, and signs of morphological improvement, were observed. The tissue sections were very close to normal and the Sertoli cells showed significant improvement. Basal membrane and spermatogenic series cells were found to be close to normal. The JTBS results showed that the application of CAPE together with CP reduced the damage caused by CP. The comet assay findings showed that CP-induced DNA damage in the spermatozoa was reduced by the administration of CAPE. In addition to CAPE treatment in CP-induced testicular injury, an increase in SOD and CAT activity ​​and the TES in the blood serum were observed, which may be evaluated as indications of improvement, although not statistically significant. Different and higher doses of CAPE could make the biochemical parameters more meaningful.

As a result, it was determined that CP affects many organs and causes serious histopathological damage in testis tissue. To prevent this damage, it was shown that CAPE, which was given for protective purposes, could be protective and corrective in the testis. It is believed that different and higher doses of CAPE could further reduce the damage.
